# Genomics Virtual Laboratory: A Practical Bioinformatics Workbench for the Cloud

**DOI:** 10.1371/journal.pone.0140829

**Published:** 2015-10-26

**Authors:** Enis Afgan, Clare Sloggett, Nuwan Goonasekera, Igor Makunin, Derek Benson, Mark Crowe, Simon Gladman, Yousef Kowsar, Michael Pheasant, Ron Horst, Andrew Lonie

**Affiliations:** 1 Victorian Life Sciences Computation Initiative (VLSCI), University of Melbourne, Melbourne, Victoria, Australia; 2 Department of Biology, Johns Hopkins University, Baltimore, Maryland, United States of America; 3 Centre for Computing and Informatics (CIR), Rudjer Boskovic Institute (RBI), Zagreb, Croatia; 4 Research Computing Centre, University of Queensland, Brisbane, Queensland, Australia; 5 Queensland Facility for Advanced Bioinformatics (QFAB), University of Queensland, Brisbane, Queensland, Australia; CNRS UMR7622 & University Paris 6 Pierre-et-Marie-Curie, FRANCE

## Abstract

**Background:**

Analyzing high throughput genomics data is a complex and compute intensive task, generally requiring numerous software tools and large reference data sets, tied together in successive stages of data transformation and visualisation. A computational platform enabling best practice genomics analysis ideally meets a number of requirements, including: a wide range of analysis and visualisation tools, closely linked to large user and reference data sets; workflow platform(s) enabling accessible, reproducible, portable analyses, through a flexible set of interfaces; highly available, scalable computational resources; and flexibility and versatility in the use of these resources to meet demands and expertise of a variety of users. Access to an appropriate computational platform can be a significant barrier to researchers, as establishing such a platform requires a large upfront investment in hardware, experience, and expertise.

**Results:**

We designed and implemented the Genomics Virtual Laboratory (GVL) as a middleware layer of machine images, cloud management tools, and online services that enable researchers to build arbitrarily sized compute clusters on demand, pre-populated with fully configured bioinformatics tools, reference datasets and workflow and visualisation options. The platform is flexible in that users can conduct analyses through web-based (Galaxy, RStudio, IPython Notebook) or command-line interfaces, and add/remove compute nodes and data resources as required. Best-practice tutorials and protocols provide a path from introductory training to practice. The GVL is available on the OpenStack-based Australian Research Cloud (http://nectar.org.au) and the Amazon Web Services cloud. The principles, implementation and build process are designed to be cloud-agnostic.

**Conclusions:**

This paper provides a blueprint for the design and implementation of a cloud-based Genomics Virtual Laboratory. We discuss scope, design considerations and technical and logistical constraints, and explore the value added to the research community through the suite of services and resources provided by our implementation.

## Introduction

### What is the problem?

Modern genome research is a data-intensive form of discovery, encompassing the generation, analysis and interpretation of increasingly large amounts of experimental data against catalogs of public genomic knowledge in complex multi-stage workflows [1]. New algorithm and tool development continues at a rapid pace to keep up with new ‘omic’ technologies [2], particularly sequencing. There are many visualisation options for exploring experimental data and public genomic catalogs (e.g. UCSC Genome Browser [3], GBrowse [4], IGV [5]). Analysis workflow platforms such as Galaxy [6], Yabi [7], Chipster [8], Mobyle [9], or GenePattern [10] (to name a few) allow biologists with little expertise in programming to develop analysis workflows and launch tasks on High Throughput Computing (HTC) clusters.

However, the reality is that the necessary tools, platforms and data services for best practice genomics are generally complicated to install and customize, require significant computational and storage resources, and typically involve a high level of ongoing maintenance to keep the software, data and hardware up-to-date. It is also the case that a single workflow platform, however comprehensive, is rarely sufficient for all the steps of a real-world analysis. This is because analyses often involve analyst decisions based on feedback from visualisation and evaluation of processing steps, requiring a combination of various analysis, data-munging and visualisation tools to carry out an end-to-end analysis. This in turn requires expertise in software development, system administration, hardware and networking, as well as access to hardware resources, all of which can be a barrier for widespread adoption of genomics by domain researchers.

The consequences of these circumstances are significant:

- Reproducibility of genomics analyses is generally poor [11], in part because analysis environments are hard to replicate [12];
- Tools and platforms that are able to provide best practice approaches are often complex, relying on technical familiarity with complicated compute environments [13];
- Even for researchers with relevant technical skills and knowledge, managing software and data resources is often a significant time burden [14];
- Skills training and education is often disconnected from practice, often because of the analysis environment constraints [15];
- Accessing sufficient computation resources is challenging with current data sets, and this is compounded by the trend to larger experimental data; for instance, moving from exome to genome scale analysis is a significant scalability problem in backend compute [16];
- Data management and movement is a technical challenge that affects the speed and accessibility of analysis [17]. Again, this is compounded by the trend towards larger data sets.

We argue that lack of widespread access to an appropriate environment for conducting best-practice analysis is a significant obstruction to reproducible, high quality research in the genomics community; and further, transitioning from training to practice places non-trivial technical and conceptual demands on researchers. Public analysis platforms, such as Galaxy, provide solutions to some of these issues (particularly accessibility), but are generally handicapped by rapid growth in per-user demand for compute resources and data storage, and the enforced constraints on flexibility that are a requirement of a centrally managed resource.

### What is the solution?

A ‘virtual laboratory’ environment to support genomic researchers that would meet a number of criteria, ideally providing:

1. Reproducibility: through workflows and stable underlying analysis platform;
2. Accessibility: through ease of gaining access to and using the platform;
3. Flexibility: by imposing as few constraints as possible in the types of analysis and the methods that may be implemented, supported via a user-controlled environment;
4. Performance: through scalability and highly available compute resources;
5. Consistency: a common platform from training to best practice;
6. Capability: through pre-population with best practice tools and reference datasets.

### The objective of building such an environment is to make a platform embodying each of these characteristics widely available to a diverse range of users, facilitating widespread best practice training and analysis for genomics.

This is, of course, not a trivial objective to achieve, as each of these criteria has significant design and technical implications:

**Reproducible** genomics requires, at a minimum, a way of accessing the same tools and reference datasets used in an analysis, combined with a comprehensive record of the steps taken in that analysis in the form of a workflow, in sufficient detail to reliably produce the same outcome from the same input data, assuming a deterministic analysis [18]. At the most basic level reproducibility can be achieved with shell scripting and documentation, but issues in ease of use, maintenance and genuine reproducibility are well-known [19], [20]. This has catalysed a number of efforts in developing platforms for reproducible scientific analysis through structured workflows, including Galaxy, Yabi, Chipster, GenePattern and numerous commercial products (e.g., Igor [21], BaseSpace (https://basespace.illumina.com/), Globus Genomics [22]). An environment supporting reproducible genomics requires at least a workflow platform and a system for ensuring stability of the underlying software and data [23].

We would define an **accessible** environment as one that is:

1. Simple to invoke or obtain access to (low cost of entry)
2. Simple to communicate with (easy to connect, low latency)
3. Simple to interact with, requiring minimal training in order to use effectively (intuitive)

Simplifying access to an analysis environment, then, requires the provider to furnish an intuitive platform that requires minimal client-side configuration—ideally, a web browser—and, further, does not require significant preparation or resources to invoke. In other words, the ideal accessible environment is one which a new user can immediately connect to and start using for training or data analysis. In many ways, public analysis services such as the Galaxy Main server (https://usegalaxy.org) and GenePattern (http://genepattern.broadinstitute.org) provide exactly this experience, and taken in isolation, meet the challenge of reproducible and accessible analysis extremely well.

However, managed services, while highly accessible, cannot provide great **flexibility**, which we would define as the freedom to both configure an environment and access that environment through a variety of means. Maximising flexibility implies user-level administrative control (e.g., configuring data, tools and, potentially, the supporting operating system directly), which is not generally possible in a centrally managed service. Hence, flexibility is in some ways the natural enemy of a managed service.

Building an analysis environment that guarantees good **performance** for a wide user base is especially challenging. In the case of a managed service for genomics, the more successful the service is in attracting users, the more likely it is that performance will suffer due to the number of users, particularly as those users explore larger data sets through a wider range of analysis options [24]. Good performance on a per-user basis is a combination of available resources, user access to those resources, underlying infrastructure limits and bottlenecks (for instance, disk I/O), and the inherent scalability of the environment. We would argue that performance in the context of a widely available, flexible genomics environment requires high-availability, scalable back-end compute resources. We will discuss performance design principles and implications in more detail in a later section, as this is a particularly challenging but critical characteristic of an environment that aims to support large genomics data analysis.

Providing a **consistent** experience from training to practice is a combination of (at least) accessibility, performance, and flexibility. Ideally an analysis environment would be accessible for new users, with training materials that follow best practice protocols delivered in an intuitive way, leading to seamless scale-up for analysis of real data sets using the same interaction paradigm and maintaining good performance.

As users become more sophisticated in genomics analysis, they often move from a single intuitive analysis platform (such as Galaxy) to multiple platforms (R, command line, custom scripts) that provide more **capability** and flexibility (generally at the expense of simplicity). Therefore, a design principle for a general genomics environment should be for that environment to be able to be used for training (implying at least an *accessible* platform), but able to scale in *flexibility* by adding more options for interaction (such as command line and/or programmatic interfaces), and scale computationally to provide the *performance* for real data analysis. For all levels of the environment, we would provide high *capability* through access to best practice tools and availability of reference datasets, and ideally linked to low latency visualisation and data interpretation services.

Table 1 summarises the above discussion and captures core implications for each category of a powerful genomics laboratory.

**Table 1:** A summary of the criteria that would define a general genomics workbench environment, and suggested implications on technical requirements.

Criteria	Design implication	Technical implication
Accessible	Minimal client-side requirements	Web based tool and management interfaces; easy start-up.
Reproducible	Workflow support + software & tool management process	Reusable, exportable workflows + automated process for deploying environment components.
Performance	User-managed scaling of compute resources; high availability resources at back end	Cloud-based architecture + interface for managing resources.
Flexible	User configurable, user administrable, command line access	Per-user instances accessible through web and command line; user-administrable environment.
Consistent	Single platform from training to analysis with layered interfaces	Tutorials and guides for training using best practice tools; protocols outlining real-world analysis steps.
Capable	Pre-populated with suite of tools for common use cases + required reference data + visualisation options	Comprehensive pre-configured tools and data; range of analysis interfaces; automated build process to implement and test complex underlying images; low latency local visualisation options.

### Wide Availability

Designing a flexible, accessible, reproducible, high-performing environment to be widely available to a large, potentially geographically dispersed audience, places serious demands on system design and architecture. One useful interpretation of ‘widely available’ is that the environment has a low cost of entry as a whole—that is, minimal preparations and resources are required before obtaining access to an analysis environment that is genuinely useful. The more obstructions that are placed before a user can start doing analysis, the less available the environment can claim to be. For example, managed public web services have a very low cost of entry and are certainly widely available. In order to make a more flexible, high performing environment widely available for open ended data analysis, we need to enable a user to quickly and intuitively build or deploy an environment that uses infrastructure resources that are relatively simple for the user to obtain.

To that end, resources underpinning the analysis environment must be low-cost, scalable, and available to the user; to provide flexibility and performance, the user must have some control of these resources. A prominent infrastructure paradigm that fits these requirements is cloud computing [25]. Cloud computing has demonstrated its suitability for providing highly available, accessible computational infrastructure suitable for data analysis [26], [27]. In the cloud model, one rents computing resources in the form of virtual machines on an as-needed basis, from a pool of resources that is large enough to guarantee high availability, and therefore good scalability.

Further, providing an environment for a large audience over a large geographic region means that network bandwidth may become an important factor in getting data to and from the environment, as bandwidth often correlates with distance from a service [28]. Thus in practice, making a flexible, high performance environment largely depends on availability of low-cost, infrastructure that is relatively close in terms of latency/bandwidth. Cloud computing often addresses this requirement through regional geographic hubs.

### Cloud Computing Solutions for Genomics

Cloud resources have become quite popular in the form of public clouds (e.g., Amazon Web Services (AWS), HP Cloud, Google Compute Engine) where one pays only for the resources consumed. These resources are provisioned as ‘bare bones’ machines that need to be carefully tailored for use in genomics. This includes procuring the required resources, installing and configuring the necessary software, and populating it with appropriate data—all tasks that are time consuming and require significant technical expertise. Consequently, a range of cloud management software applications have been developed that tailor cloud resources to fulfill a functional role in bioinformatics. In addition to these dedicated, cloud-aware, applications, a number of platforms or virtual laboratories have also been developed that aggregate the functionality of many applications. *Galaxy on the Cloud* [29] offers a preconfigured Galaxy application in a cloud environment. More generally, Globus Galaxies [30] offers a general purpose platform for deploying software-as-a-service solutions in the cloud based on Galaxy. Additional platforms that focus on Big Data solutions and use of the MapReduce model include Cloudgene [31] and Eoulsan [32]. See Calabrese and Cannataro [33] for a more details overview of the existing cloud-aware applications and platforms.

Over the past few years, there has been an increasing trend towards cloud resources also becoming available as research infrastructures, for example the Open Science Data Cloud in the US, EGI Federated Cloud in the EU, and NeCTAR Research Cloud in Australia. These provide alternatives to the public clouds by offering centralized access to clouds for researchers and projects, generally with merit-based allocation as opposed to direct financial expense to the researcher. NeCTAR, for example, offers access to an OpenStack-based Research Cloud (http://nectar.org.au/research-cloud) where any researcher in Australia can access limited virtual machines and storage, and apply for larger allocations of both.

These national compute infrastructures provide readily available virtual hardware, with the opportunity to address the scalability issue both at the personal level (as a researcher can request temporary resources as required) and at the community level (as each research group can apply their own merit-allocated CPU and storage quota, rather than overburdening a centralised server). The advent of research-oriented cloud computing has created an opportunity to build support for bioinformatics analyses on these highly available national infrastructures and public clouds.

## Results and Discussion

### Designing the Genomics Virtual Laboratory

In this section we provide a template for designing and building a genomics analysis environment based on a cloud-aware workbench of genomics tools platforms.

In response to the described circumstances, we developed the Genomics Virtual Laboratory (GVL). The GVL is designed to be a comprehensive genomics analysis environment supporting accessible best practice genomics for as wide a community of researchers as possible; this philosophy directs the design and implementation of the GVL to a great extent, as accessibility, flexibility, performance and wide availability are principal drivers. **In practice, the GVL is a combination of scalable compute infrastructure, workflow platforms, genomics utilities, and community resources.**

The primary objective of the GVL is to enable researchers to spawn and/or access automatically configured and highly available genomics analysis tools and platforms as a versatile workbench. Workbench instances may be centrally-managed servers or standalone and dedicated cloud-based versions. Either option is scalable and comes pre-populated with field-tested and best-of-breed software solutions in genomics, increasing reproducibility and usefulness of the solution. The aim is to offer a true genomics workbench suitable for bioinformatics data analysis for users with a variety of needs.

The design principle for the GVL has been to attempt to meet each of the design criteria—accessibility, reproducibility, flexibility, performance, capability and consistency—using existing software as much as possible. There are already very mature genomics workflow platforms providing accessibility and reproducibility, for instance; likewise, sophisticated platforms for flexible programmatic and statistical approaches to analysis and visualisation. With that in mind, a number of design choices were made on the functional software components of the GVL. These are summarised in Table 2, while individual solutions are described in more detail under *Using the Genomics Virtual Laboratory*.

**Table 2:** Summary of specific technical solutions used to meet the design requirements of the GVL.

Criteria	Technical implication	Technical solution
Accessible	Web-based tool and management interfaces; easy start-up	*Galaxy; RStudio Server; IPython Notebook; in-browser remote desktop; CloudMan; Cloud cluster launcher; integrated GVL Dashboard*
Reproducible	Reusable, exportable workflows + automated process for deploying environment components	*Galaxy; IPython Notebook; GVL build process; CloudMan*
Performance	Cloud-based architecture + interface for managing resources	*CloudMan (with Slurm job manager)*
Flexible	Per-user instances accessible through web and command line; user-administrable environment	*CloudMan; SSH access; GVL command line utilities; Galaxy web-based admin + Galaxy Toolshed integration*
Consistent	Tutorials and guides for training using best practice tools; protocols outlining real-world analysis steps.	*Tutorials; established workflows*, *protocols*
Capable	Extensive pre-configured genomics software tools and data; range of analysis interfaces; automated build process to implement and test complex underlying components; low latency local visualisation options	*GVL build process; managed services*, *including local UCSC browser; shared reference datasets*

The choice of the components were based on a number of factors, including platform functionality, platform maturity, community uptake and complementarity (e.g. Galaxy is focussed on bioinformatics workflows and easy access to tools; IPython Notebook [34] on programmatic analyses; RStudio Server (http://www.rstudio.org/) on statistical analyses; UCSC genome browser is perhaps the most popular genome browser). In the case of a decision on management middleware for deploying the platforms, CloudMan [35] has been demonstrably successful in providing cloud-based genomics workflow platforms based on Galaxy [29] and was therefore the software of choice for this role. Additionally, local expertise was a factor in the final design decisions of the GVL workbench (e.g., tutorials).

Also considered in designing the GVL were the advantages of different sections of the community. As a scalable, extensive and customisable framework, the GVL primarily caters to the *individual genomics researchers and small labs*—it offers a simple, quick and reliable method for obtaining access to a scalable environment for genomics analysis and visualisation. It allows complete control over self-deployed instances and easy access to common data resources, such as indexed genomes, which can otherwise be problematic and time consuming to obtain. Finally, it offers substantial resources for learning new analysis methods.

Next, the GVL caters to the *broader bioinformatics community*—it provides a low-cost-of-entry target platform for collaborative genomics analysis and data sharing that is based on a common platform. The ability to customize the platform further facilitates its use for tool development and distribution. These features together also make the GVL a good environment for developing training materials and curricula, and for teaching. Because it is based on tools that intrinsically enable a reusable record of any analysis (e.g., Galaxy, IPython Notebook), the GVL also encourages reproducible research.

Finally, the GVL appeals to *research infrastructure bodies and research institutions* because it promotes democratized access to large scale, complex genomics analysis infrastructure. It focuses on simple and cost effective scaling (both in breadth and depth) of national computational infrastructure by delivering accessible and powerful solution to genomics researchers.

### Using the Genomics Virtual Laboratory

In this section we describe the resulting functionality of the GVL from a user perspective—that of an ordinary end-user carrying out research or training, and that of a developer or a researcher building new tools and infrastructure.

Using the GVL as a researcher. From the users’ perspective, the GVL comprises three main parts: the cloud-launchable GVL workbench, always-on managed services, and community resources.

**Fig 1:**
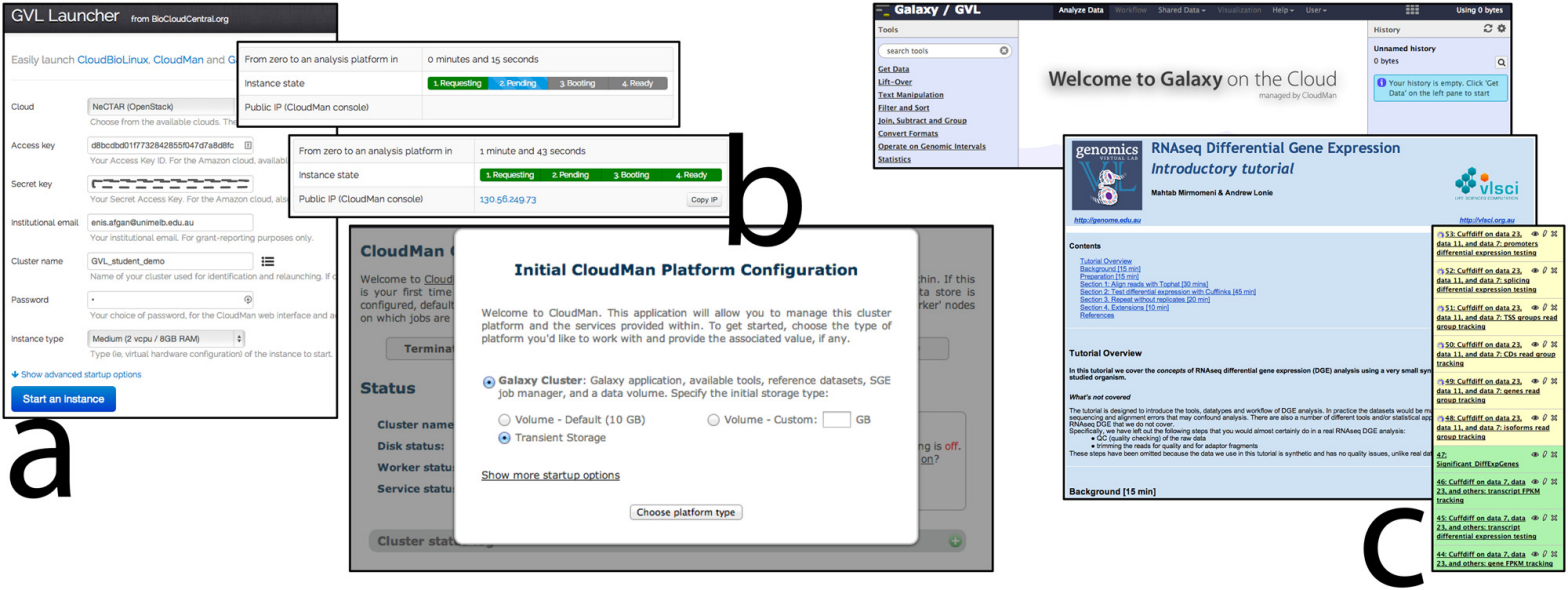
The GVL launch process for starting self-launched instances of the GVL workbench. **(a)** A user initiates the launch process via the launch service (*launch*.*genome*.*edu*.*au*) by providing their cloud credentials to the launcher application and **(b)** within a few minutes is able to access the management interface (CloudMan) on the deployed instance of the workbench. **(c)** After workbench services have started, the researcher can use the applications as desired (e.g., Galaxy).

**Fig 2:**
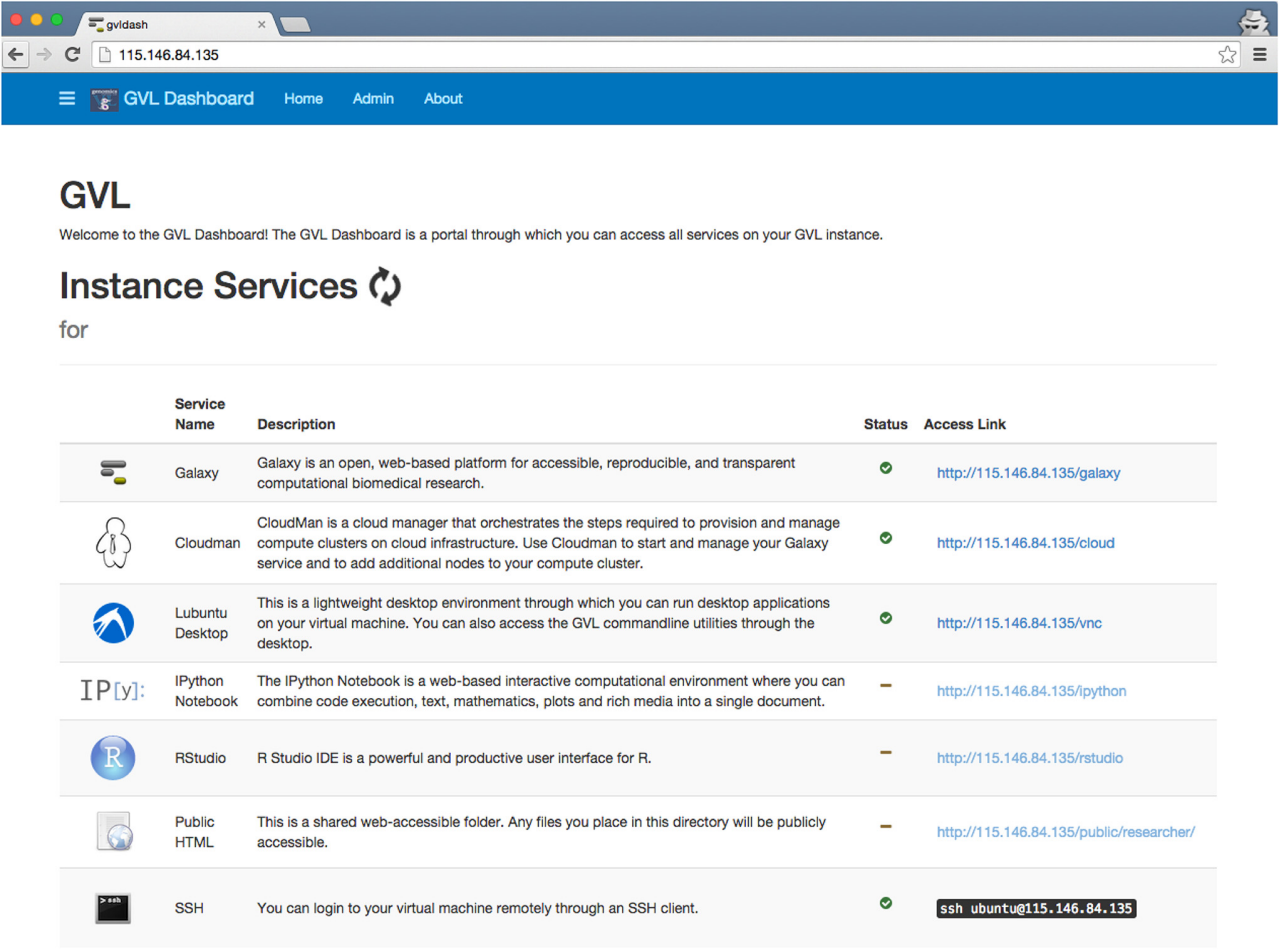
A screenshot of the GVL Dashboard. The GVL Dashboard is a portal running on every GVL instance. It lists all of the available services, their status, and offers a direct link to access those.

**Cloud-launchable instances**: these are based on the GVL machine image, and can be easily launched and configured via a launcher web application (Fig 1). Each launched instance runs the following services:

- *The GVL Dashboard*: provides easy access to all the services and their status—this is the default landing page for all self-launched GVL instances (Fig 2);
- *CloudMan*: used to manage cloud services associated with an instance, such as compute and storage. This includes the ability to scale the instance by adding worker nodes, turning each GVL instance into a virtual cluster-on-the-cloud;
- *Galaxy*: is a popular web-enabled platform for reproducible bioinformatics analysis, which is capable of deploying jobs over the cluster-on-the-cloud. Researchers can customise Galaxy via the Galaxy Toolshed [36] and Galaxy Data Managers [37];
- *RStudio Server*: a web-based platform for statistical analysis using R;
- *IPython Notebook*: a web-based platform for programmatic analysis using Python;
- *VNC Remote Desktop*: a web-based remote desktop interface to the Linux operating system (Ubuntu);
- *An underlying Linux environment* with full *ssh* access and administrative control. This includes access to command-line bioinformatics tools and reference data that comes preinstalled on the system (i.e., all the tools installed via Galaxy).

**Managed services**: these are services hosted by the GVL project that are readily available to anyone:

- *Galaxy Tutorial instance*: a managed Galaxy tutorial server (called Galaxy-tut; available at *galaxy-tut*.*genome*.*edu*.*au)* tailored for training and interactive learning with all the tools required to run GVL Tutorials. This server can be freely used by any researcher for training purposes, including running GVL tutorials and workshops;
- *Galaxy Research instance*: a managed Galaxy server for real-world research analyses (called Galaxy-QLD; available at *galaxy-qld*.*genome*.*edu*.*au*) that offers a broad spectrum of tools and generous storage quotas (currently 1TB);
- A local mirror of the UCSC genome browser (available on the Australian Research Cloud at *ucsc*.*genome*.*edu*.*au*) and associated visualisation services for fast access to hosted data;
- *Shared reference datasets*, such as reference genomes and indices, that are automatically made available to any launched GVL instance as a read-only networked file system.

**Community resources and support** in the form of comprehensive online teaching materials around common genomics experimental analyses, supported with a mechanism of delivering those to the bioinformatics community:

- *GVL Tutorials*: introduce important bioinformatics analysis techniques to new users. These tutorials are self-contained and can be self-driven or used in a workshop setting. For the most part, they make use of Galaxy for its excellent learning environment, and can be run on a training instance such as Galaxy-tut, or on a self-launched instance. Developed tutorials are based on common best practices or published methods (e.g., Trapnell *et al*. [38]);
- *GVL Protocols*: are field-tested procedural methods in the design and implementation of a bioinformatics analysis, which, in comparison to Tutorials, provide less detailed instructions on each step, but more advice on analysis options and best-practice principles. Protocols include a general overview of the problem and a skeleton for an analysis but do not specify exact tools, parameters, or sample data. Consequently, they are seen as a roadmap for an analysis that should be extended or modified to accommodate the needs of a particular research analysis;
- *Galaxy-enabled tools built by the GVL team*: developed tools are available through the main Galaxy Toolshed and come pre-installed on any launched GVL instance. Many tools are used in GVL Tutorials and Protocols;
- Email-based helpdesk support for all components of the GVL.

These resources are presented to users as three broad categories, LEARN, USE and GET, which may be familiar to Galaxy users from http://galaxyproject.org/:

- USE—make use of managed services, including Galaxy servers and the UCSC genome browser;
- GET—get your own analysis platform using cloud infrastructure, with full administrative control and additional power-user utilities. This option allows a user to transition smoothly from training to research—a user-launched GVL instance provides a research environment consistent with the USE and LEARN environments, but allows researchers full control for further customisation (Fig 1);
- LEARN—learn bioinformatics analysis using GVL Tutorials, running them either on the Galaxy-Tut server or on a user's own instance. More advanced users can make use of GVL Protocols.

Currently, the GVL is implemented and available on the Australian Research Cloud as well as the Amazon Web Services (Sydney region). In addition, managed services (i.e., USE—running on the Research Cloud) and products (i.e., LEARN) are freely available to anyone. The self-launched instances (i.e., GET) are available to the Australian researchers and groups that have an allocation on the Research Cloud or anyone with Amazon Web Services credentials. All of the GVL services are linked from the GVL main webpage: https://genome.edu.au.

Leveraging the GVL as a developer. From the technical development perspective, the GVL comprises:

- A set of machine images, cloud resource components, and source code repositories containing the functional elements of the workbench: Galaxy, IPython Notebook, RStudio, bioinformatics toolkit;
- A sophisticated cloud infrastructure and application management system (i.e., CloudMan) to:
  - Enable users to launch/deploy a new instance of the workbench;
  - Manage workbench services and resources as required; and
  - Scale the backend cloud infrastructure to match performance requirements, by building a cluster-on-the-cloud with Slurm [39] as the job manager.
- Access to a shared file system containing large reference data; this file system is geographically replicated via GlusterFS file system (http://www.gluster.org/) and any launched instance can connect to it in read-only mode;
- An automated process for generating machine images and other cloud components that are pre-populated with the latest tools. The build process is based on a set of Ansible roles (http://www.ansible.com/) that are publicly available as a set of open source scripts (https://bitbucket.org/gvl/gvl-image-playbook). The GVL build process is compatible with multiple clouds and can be used to replicate the GVL environment by anyone (for documentation on how to do this, see the mentioned source code repository). Ansible roles are not used by ordinary end-users, who make use of a pre-built image to launch their GVL instance. However, they are useful for developing new images or deploying to new cloud environments.

A user-deployed GVL instance provides a Linux environment pre-populated with common programming languages and bioinformatics libraries, as well as with popular analysis platforms. A GVL instance also comes with a preconfigured database server (PostgreSQL), cluster management system (Slurm), and with CloudMan, which is capable of adding and removing worker nodes from the virtual cluster, managing various storage options, and managing GVL services. This environment provides developers and bioinformaticians with a convenient platform for tool development and testing, both command-line and Galaxy-based. The choice of type of machine instance and the cluster-on-the-cloud scalability features also provides an excellent environment for tool benchmarking and scalability testing.

The open build system of the GVL, and the general applicability of the cluster-on-the-cloud and service management model, make the GVL a good starting point for the development of other cloud-based research environments. Labs or developers can thus take the core GVL and customise it or extend it to meet their particular needs. This capability has already been exploited within the GVL project itself: genomics researchers often work in a specific sub-domain, each of which requires specific set of tools. Using the GVL's flexible build system, we are developing specialised "flavours" of the GVL workbench suitable for particular uses. The following flavours are currently under development and more are planned (in addition, community-contributed flavours are welcomed):

- *Full*: a complete toolset deployed by the GVL
- *Tutorial*: a set of tools used by the GVL tutorials
- *Microbial*: a toolset focused on microbial data analysis

The available flavours are selectable for self-launched instances via the Launcher app.

### End-to-end usage scenario

Thus far we have described the GVL in terms of its components. In this section we describe an end-to-end usage scenario, illustrating how the GVL can support a user from training through to full analyses.

In our experience, a relatively complex but commonly requested analysis is RNA-seq based differential gene expression analysis (DGE). This use-case consists of a number of processing steps, and has a variety of tools options, published guides for best practice, and visualisation requirements that again can be met with myriad options. Aspects of this use-case are well established and can be implemented as runnable workflows; other aspects require researcher or analyst input for interpretation. In this scenario, we envision a biologist, or a bioinformatician new to RNA-seq analysis, who wishes to learn how to conduct such analyses well and to apply them to their own data.

Differential gene expression analysis aims to discover genes whose expression level varies under the experimental conditions of interest [38]. RNA-seq has been shown to allow high technical accuracy for such analyses relative to older microarray-based methods [40]. Due to the constraints imposed by the large number of genes in most organisms, and the relatively small number of samples that can feasibly be included in most studies, increasingly sophisticated statistical methods have been developed to take advantage of observed statistical properties of gene expression [41,42]. These methods may be available to researchers as command-line tools or as libraries for programmatic analysis, particularly in R [43]. Both popular command-line tools, and R libraries, have been made available through Galaxy. These tools are also available via the GVL command-line and GVL RStudio, with the latter allowing maximum flexibility in developing more complex analyses.

In this scenario, we restrict ourselves to differential analysis of gene expression, and do not discuss the many other types of analysis that may be carried out with RNA-seq data. A typical RNA-seq DGE analysis consists of the following steps:

1. Begin with RNA-seq data from a high-throughput sequencing experiment, usually in the form of FASTQ files. Currently, a typical amount of data for this analysis is on the order of 20 million reads per sample, where current read lengths are likely to be 100-150bp, giving 2-3GB of raw data per sample. Usually, in order to perform a statistically robust analysis, multiple samples from each experimental condition are required, giving data on the order of ~10-15GB.
2. Align sequence reads to reference genome. This step can be carried out using well-established tools, but is compute and I/O intensive.
3. Count aligned reads against gene model to produce table of gene feature counts per sample.
4. Statistically test for differential expression of gene features between groups of samples. This step may be carried out using relatively simple methods or more advanced statistical approaches. More advanced approaches allow researchers to handle complex experimental designs.
5. In many cases, the project will involve further analysis, such as pathway or gene set enrichment analysis, to help interpret the significance of the differentially expressed genes.

In this scenario, the GVL allows a novice to go through the following processes:

1. The researcher learns the concepts of RNA-seq differential analysis through the GVL *Introductory RNA-seq* tutorial (https://genome.edu.au/wiki/Learn), which guides them step-by-step through the workflow and concepts. This tutorial takes researchers from the original read sequences, through read alignment, read-counting, and basic statistical analysis. Researchers can make use of the managed Galaxy-tut service (https://galaxy-tut.edu.au/) to work through this tutorial. Alternatively, researchers may launch their own personal instance to use for these tutorials (see Fig 1). For this step researchers may also wish to take advantage of introductory RNA-seq workshops run by the institutions supporting the GVL project, or the Galaxy Training Network (https://wiki.galaxyproject.org/Teach/GTN). These are usually one-day, hands-on workshops that include an introduction to launching an instance on the cloud, an introduction to using the Galaxy platform, and the *Introductory RNA-seq* tutorial.
2. The researcher applies these analysis techniques to their own data. This analysis is enough to give preliminary results on real data, and concrete understanding of the method. For this step the researcher may use either the same personal instance as in Step (1), or a larger managed service. If the project is particularly large and merits its own compute allocation, researchers will be able to obtain Research Cloud quota from the NeCTAR Research Cloud and launch larger cloud instances.
3. The researcher learns more advanced DGE techniques and concepts through the GVL *Advanced RNA-seq* tutorial (https://genome.edu.au/wiki/Learn). This tutorial applies alternative and more advanced statistical analysis packages. These approaches can still be accessed via the Galaxy interface via, for instance, a Galaxy wrapper around a standard edgeR-based analysis [44]. In most cases, at this point the researcher is in a position to obtain publication-quality results on their data.
4. Researchers may optionally move to RStudio or IPython Notebook on their GVL instance to produce more flexible visualisations of their results, or as a means to access downstream analysis tools appropriate to the project.

In some cases the experimental design may be particularly complex and require advanced understanding of the statistical issues involved. In such cases, there is no real substitute for statistical expertise, and the researcher or a collaborator on the project should have this. In this case our researcher can move to their GVL instance's RStudio platform in Step (3), which gives them a more advanced and flexible set of statistical tools. It is possible to carry out computationally-intensive steps (alignment of reads) in Galaxy or via the command-line, and then access the resulting gene counts in RStudio. Most popular Bioconductor libraries for DGE analysis are pre-installed into R on GVL instances.

The results output from any of these analyses can be stored in persistent cloud storage and/or downloaded for further use in other tools. The researcher can shut down their instance and use CloudMan functionality to re-initialise the same environment at a later date, or to share the complete workbench specification with other researchers [45].

The analysis steps themselves along with the data can be stored and published as a Galaxy History. In the case of a more advanced analysis, the steps can be stored and published as an R or R-markdown script [46], or an IPython Notebook document. All of these exported analyses have the potential to be imported into another GVL instance and re-run, providing excellent reproducibility. The full web and command-line access to the GVL platform means that the researcher and their collaborators are free to move onto any advanced methods appropriate to their project.

## Conclusions and Future Work

**Fig 3:**
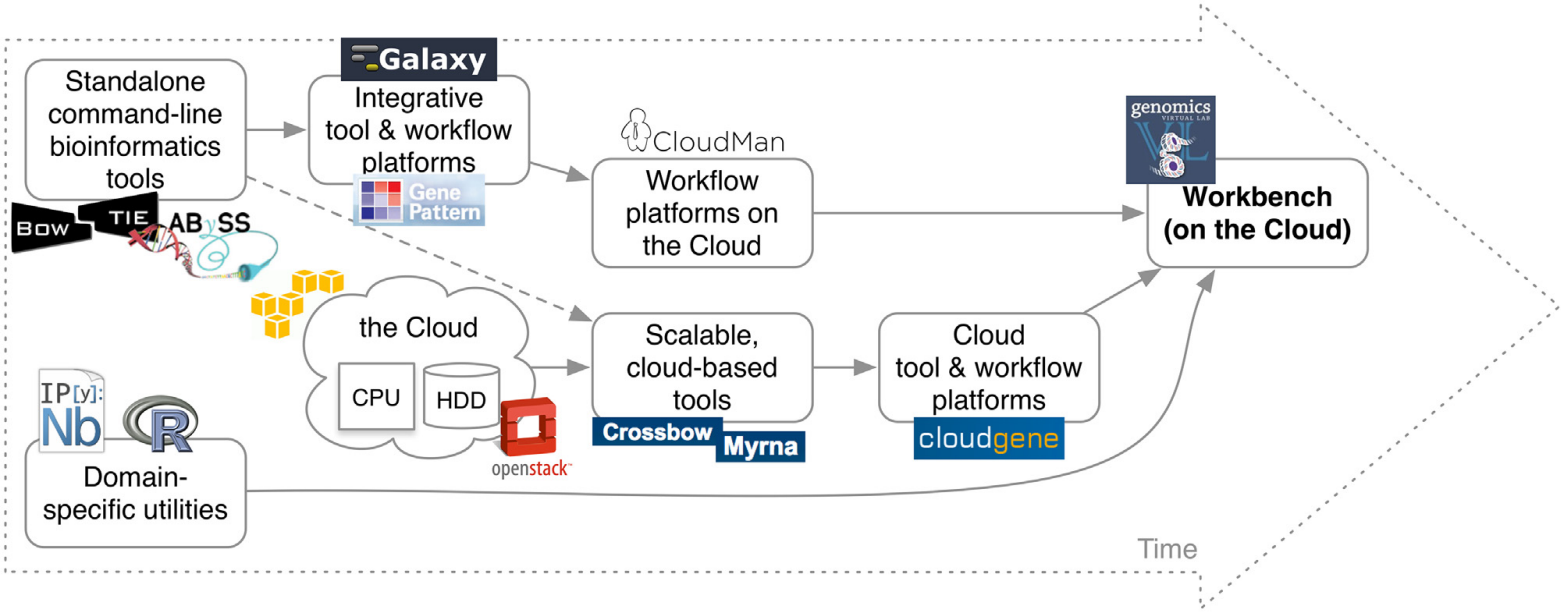
An evolution of the data analysis solutions for genomics. Initially, standalone and purpose-specific tools were most prevalent. As the complexity of analyses grew, new platforms formed that aggregate many standalone tools and support different types of computational infrastructures to offer more versatile functionality. The GVL represents another step in this evolution where it aggregates a large number of the best-of-breed software and technologies available today.

Driven by a landscape of rapidly evolving data-generating technologies, the field of genomics has quickly grown into a demanding domain requiring a complex ecosystem of tools, technologies, computers, and data—all honed to support multi-step pipelines using dozens of tools and dealing with gigabytes of data. The reality is that processing research-sized genomic data requires a comprehensive data analysis workbench (Fig 3). This in turn inflicts a high level of maintenance overhead and required technical expertise on the data analysis process, which is a barrier to entry for biology-focused researchers.

The GVL has been established to reduce this barrier by improving understanding and accessibility of bioinformatics tools, backed by accessible, scalable and robust computational infrastructure. The GVL provides a set of well-rounded services that are accepted throughout the community and supports activities ranging from teaching and training to end-to-end research analysis, making it applicable as a bioinformatics workbench and a computational platform not only in technical terms but also in terms of a community that it supports. Services unified by the project supply a much-needed locus of tools and technologies allowing researchers to more easily and readily explore the data analysis space. GVL’s services are simple to access via the project’s website (genome.edu.au).

Ultimately, the GVL represents a blueprint for deploying virtual laboratories, even for domains other than genomics: it defines the components required to establish a virtual laboratory, technologies to embody these components, and use-cases to deliver a purposeful product. It supports the notion of Science-as-a-Service [47] and can be used as a validated, exemplar method in the future. The GVL's design pattern and build system are currently being exploited to deploy the GVL onto other cloud stacks, and to develop customised "flavours" of the GVL for specific research sub-domains.

## Methods

### Building the Genomics Virtual Laboratory

In this section we describe the broad technical details of how the GVL is built.

The GVL is implemented by composing a carefully selected set of state-of-the-art software that has seen wide adoption and demonstrated utility in the space of bioinformatics data analysis. Much of the effort in building the GVL workbench has been a very significant technical effort in developing architectural approaches to aggregate and automate the process for generating the functional services of the workbench, and developing and extending sophisticated management software that allows users to first deploy and then scale and manage the resources and services underpinning the workbench. The developed components were created to be reusable, the outcome being that the GVL workbench can be replicated on any compatible cloud. Parts of the workbench stack are also sufficiently generic to be repurposable in contexts other than a genomics workbench (e.g., CloudMan as a generic cluster-in-the-cloud).

**Fig 4:**
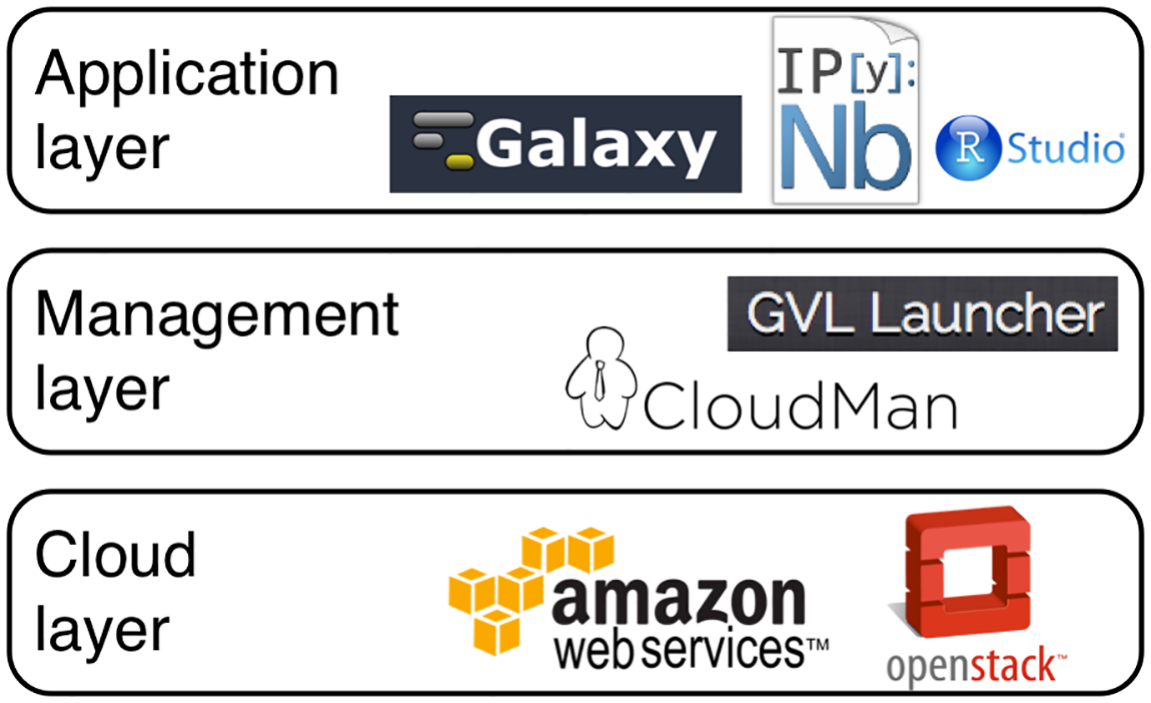
Three basic architectural layers composing the GVL workbench. The GVL leverages cloud resource and is compatible with multiple cloud technologies. Through a set of cloud resource management tools, the details of cloud resources are hidden enabling non-cloud aware applications to readily execute in this environment.

Architecturally, the GVL is composed of three broad layers: the Cloud as the base layer that offers resource capacity; the middle layer that provides resource management, structure and control over the cloud resources; and the application layer that contains the tools, utilities and data in an accessible form (Fig 4).

In the context of the GVL implemented in Australia, the GVL relies on the NeCTAR Research Cloud as the base layer. The GVL image is also available for launch on the AWS Cloud, in the Sydney region. Cloud resources provide a uniform and readily available compute infrastructure. Technologies underpinning the GVL were designed to be cloud-agnostic and rely features common to a set of Infrastructure-as-a-Service clouds [48] (also see below). Combined with the automated build process, this approach makes it feasible to deploy the GVL on a range of clouds available around the world.

The middle layer is primarily handled by CloudMan, which, as part of the GVL, needed to be extended to support clouds other than AWS [49]. As described throughout the text, CloudMan creates a virtual cluster-in-the-cloud. The created cluster can behave as a traditional cluster environment, permitting applications designed for cluster environments to be readily run. Hence, no modification to the top level applications is necessary. The GVL management layer also offers the *launch* service. This is a web application used to launch GVL instances. It initiates the provisioning of the required cloud resources on any cloud supported by CloudMan and it monitors the launch process. It was implemented in Django and the deployment process has been automated as part of the GVL build process.

Finally, the application layer is composed from all the bioinformatics software making up the GVL workbench; minimal changes were required by the GVL to the software in this layer. Along with these minor changes, some "glue" components have been added to the application layer to unify the user experience, in particular the GVL Dashboard (Fig 2 above). The GVL Dashboard is a portal that runs on GVL instances and provides an overview of the state of all application-level services running on the given instance. All of the software development performed as part of the GVL has been released into the open-source domain (https://bitbucket.org/gvl), with many of the contributions having been incorporated into the respective parent projects.

**Fig 5:**
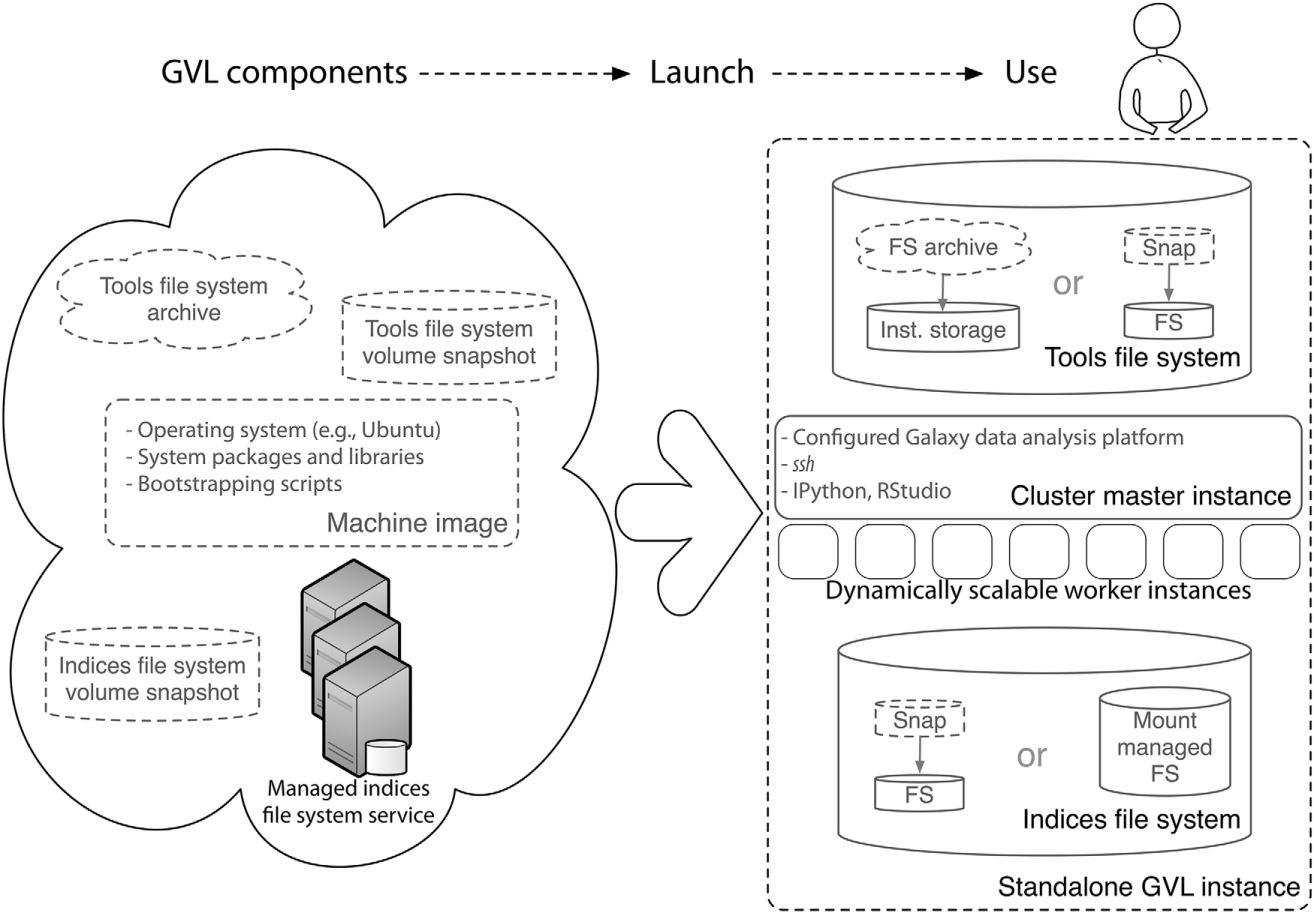
Architectural components of the GVL’s management layer. Each GVL instance is, at runtime, composed of a number of components that the GVL provides: a virtual machine image, a volume snapshot or an archive of the tools file system, and a snapshot or a hosted instance of the indices file system. Combined at runtime by CloudMan into a virtual cluster, the components enable a flexible and feature-full bioinformatics workbench.

The management layer is perhaps the aspect of the GVL with the most technical complexity, and is described in more detail now: it is comprised of a number of system-level components, including the virtual machine image, the tools file system and the indices file system. Fig 5 captures the architecture encapsulated by these components. These components are built using a set of Ansible roles that automate the build process(https://bitbucket.org/gvl/gvl-image-playbook). The architecture tying together all these individual components is what enables scalable compute at the back end and fast access to large reference datasets, making the platform of practical use for performing research-scale data analysis. This architecture is primarily implemented through the CloudMan application.

- The *machine image* represents a blueprint for the required operating system and the system packages and libraries. The machine image also facilitates the GVL launch process (initiated by the *launch* service) by allowing instance bootstrapping. Once a machine image is launched, it is considered a live instance and the bootstrapping scripts contained within initiate required runtime configuration that leads to an automatically configured cluster and a data analysis platform. As well as the machine image itself, the scripts used to build this image have been made available in the open-source domain.
- The *tools file system* contains the Galaxy application and the associated bioinformatics tools and configurations. This file system has been implemented in two alternative versions: (1) as a volume snapshot and (2) as a downloadable archive. For the volume snapshot, at instance launch time, the volume snapshot is converted (by an API request to the cloud middleware) into a volume that is then attached and mounted as a file system. The created volume is under the ownership of the tenancy (i.e., user) that created it and is persistent across cluster invocations. This means that the entire cluster with all installed applications, configurations and data can safely be shut down during the period of inactivity and later started back up with all data and configuration available in the same state as before cluster termination. This model requires the tenancy to have an appropriate volume allocation and for the volume snapshot to be available in the same cloud availability zone that the cluster is launched in. Because not all users have a volume allocation, we have also made the file system available as a downloadable archive. At instance launch time, the archive is extracted onto the instance’s transient storage with the same content as the volume-based file system. Currently, this file system is only a few hundred megabytes in size and with the colocation and replication of the data across a cloud, the time required to download and extract the archive is comparable to the time it takes to complete the request to create a volume from a snapshot. This model makes it possible to create an exact replica of the necessary file system in any cloud region using only transient instance storage. Crucially important is to realize that, although it allows the GVL to be used even by users who have no available volume storage, this model of launching GVL services is not persistent across invocations and once a cluster is terminated, the data is gone.
- The *indices file system* contains formatted reference genome index data used by a variety of bioinformatics tools (e.g., during mapping). The reference file system is updated with new reference genomes as requested by the users and is currently several hundred gigabytes in size. To facilitate reuse of this valuable resource, as part of the GVL, we have made the file system available in two formats: as a volume snapshot and as a high-performance shared file system. This shared file system is a read-only, GlusterFS-based instance of the file system that launched instances simply mount and use in read-only mode. Replicated over several zones of the NeCTAR research cloud and hosted over an average of two dedicated virtual machines, the service has shown remarkable availability and stability under load (during workshops, for example). The reference data is kept separate from the data uploaded by users or the tool installation and configuration data; such data is kept local to each instance, which ensures only the person that launched the instance has access to it. Users who wish to add their own reference data may add it in parallel to this data, or may make use of the volume snapshot option to copy the entire reference data file system into their own storage allocation.
